# Association between an Internet-Based Measure of Area Racism and Black Mortality

**DOI:** 10.1371/journal.pone.0122963

**Published:** 2015-04-24

**Authors:** David H. Chae, Sean Clouston, Mark L. Hatzenbuehler, Michael R. Kramer, Hannah L. F. Cooper, Sacoby M. Wilson, Seth I. Stephens-Davidowitz, Robert S. Gold, Bruce G. Link

**Affiliations:** 1 Department of Epidemiology and Biostatistics, University of Maryland, College Park, School of Public Health, College Park, Maryland, United States of America; 2 Department of Preventive Medicine, Stony Brook University, Health Sciences Center, Stony Brook, New York, United States of America; 3 Department of Sociomedical Sciences, Columbia University, Mailman School of Public Health, New York, New York, United States of America; 4 Department of Epidemiology, Emory University, Rollins School of Public Health, Atlanta, Georgia, United States of America; 5 Department of Behavioral Sciences and Health Education, Emory University, Rollins School of Public Health, Atlanta, Georgia, United States of America; 6 Maryland Institute for Applied Environmental Health, University of Maryland, College Park, School of Public Health, College Park, Maryland, United States of America; 7 Department of Economics, Harvard University, Cambridge, Massachusetts, United States of America; Leibniz Institute for Prevention Research and Epidemiology (BIPS), GERMANY

## Abstract

Racial disparities in health are well-documented and represent a significant public health concern in the US. Racism-related factors contribute to poorer health and higher mortality rates among Blacks compared to other racial groups. However, methods to measure racism and monitor its associations with health at the population-level have remained elusive. In this study, we investigated the utility of a previously developed Internet search-based proxy of area racism as a predictor of Black mortality rates. Area racism was the proportion of Google searches containing the “N-word” in 196 designated market areas (DMAs). Negative binomial regression models were specified taking into account individual age, sex, year of death, and Census region and adjusted to the 2000 US standard population to examine the association between area racism and Black mortality rates, which were derived from death certificates and mid-year population counts collated by the National Center for Health Statistics (2004–2009). DMAs characterized by a one standard deviation greater level of area racism were associated with an 8.2% increase in the all-cause Black mortality rate, equivalent to over 30,000 deaths annually. The magnitude of this effect was attenuated to 5.7% after adjustment for DMA-level demographic and Black socioeconomic covariates. A model controlling for the White mortality rate was used to further adjust for unmeasured confounders that influence mortality overall in a geographic area, and to examine Black-White disparities in the mortality rate. Area racism remained significantly associated with the all-cause Black mortality rate (mortality rate ratio = 1.036; 95% confidence interval = 1.015, 1.057; p = 0.001). Models further examining cause-specific Black mortality rates revealed significant associations with heart disease, cancer, and stroke. These findings are congruent with studies documenting the deleterious impact of racism on health among Blacks. Our study contributes to evidence that racism shapes patterns in mortality and generates racial disparities in health.

## Introduction

A growing body of evidence indicates that the unique constellation of environmental stressors and psychosocial challenges experienced by Blacks in the US contributes to accelerated declines in health and generates racial disparities [1]. Of these stressors, there has been increasing attention to the impact of racism-related factors, including interpersonal experiences of racial discrimination [2]. Racially motivated experiences of discrimination impact health via diminished socioeconomic attainment and by enforcing patterns in racial residential segregation, geographically isolating large segments of the Black population into worse neighborhood conditions [3]. These areas are typically characterized by social anathemas such as poverty and crime, and fewer health-promoting resources, including recreational facilities, parks, supermarkets, and quality healthcare. Such characteristics shape health behaviors such as exercise, diet, and substance use [4, 5]. Racial discrimination in employment can also lead to lower income and greater financial strain, which in turn have been linked to worse mental and physical health outcomes [6].

In addition to these indirect socioeconomic and neighborhood effects, racial discrimination may also directly impact health by engaging psychobiological mechanisms induced in the stress response [7]. Experiences of racially-motivated discrimination are inherently stressful, and may undermine psychological adjustment particularly when viewed as being outside of personal control, resulting in depression, anxiety, and anger [8]. These negative affective and cognitive responses are associated with maladaptive health behaviors and greater risk of chronic disease. Racial discrimination can also affect health by eliciting a cascade of biochemical reactions that over time can damage biological systems [9]. As a source of psychosocial stress, racial discrimination may lead to premature physiologic deterioration or “wear and tear,” ultimately compromising the ability of the body to respond to such challenges and increasing susceptibility to and acceleration of chronic diseases [10]. Research on discrimination has found that it is related to a range of biological markers of stress, including measures of oxidative stress and inflammation [11–13].

Despite this accumulating evidence, a central dilemma in epidemiologic studies is related to the measurement of racism. This research has mostly relied on self-report measures of interpersonal experiences of discrimination, which faces limitations given the motivational ambiguity that characterizes contemporary discrimination [14]. For example, although legally sanctioned forms of criminal profiling may not have an overt racial component, they have been applied inequitably and found to disproportionately impact racial minorities [15]. Racial discrimination in employment and housing markets has also been found to occur covertly despite being expressly prohibited by law [16, 17]. Discrimination can also be experienced more subtly through interpersonal interactions, such as instances of being treated with less respect or courtesy, being followed in stores, or receiving poorer service at restaurants [18]. In contrast to traditional, overtly racially motivated discrimination and prejudice, attributions of intent for these experiences are often less clear. Furthermore, individuals may not disclose experiences of discrimination, or may be unaware “invisible” discrimination and racist events that are not directly experienced [14]. For instance, not all forms of discrimination have an identifiable perpetrator, e.g., when resumes from candidates for employment or housing applicants with “Black names” are systematically rejected. [19, 20]. These measurement challenges may account for inconsistent or null findings on the relationship between health outcomes and self-reported racial discrimination, which is often influenced by subjective appraisal of the motivation for unfair treatment. In fact, results from a systematic review revealed that nearly two-thirds of studies found no statistically significant relationship between self-reported racism experiences and physical health outcomes [21].

Other studies have examined racial segregation as an indicator of institutionalized racism, which to some extent is perpetuated by discriminatory practices and the racial preferences of Whites [22]. While this line of research has provided important insights into the residential conditions experienced by Blacks, such measures as proxies for racial discrimination and prejudice are limited as they only indirectly capture racial attitudes in a geographic area.

In this study, we examined a previously developed Internet-based measure of “area racism” that did not rely on the provision of responses to survey questions and is less susceptible to social desirability bias; it may also more directly assess racial attitudes in a geographic area [23]. This measure, calculated based on Internet search queries containing the “N-word”, was strongly associated with the differential in 2008 votes for Barack Obama, the Black Democratic presidential candidate, vs. 2004 votes for John Kerry, the White Democratic presidential candidate. Studies have found that Internet searches on other subjects, including religiosity and firearms, reflect socio-demographic characteristics of the underlying population [24, 25]. For example, the percent of a state’s residents believing in God explains 65% of the variation in search volume for the word “God” [23]. Internet queries of health conditions have also been used for disease surveillance, including influenza outbreaks, and have been found to be a stronger predictor than pharmacy records [26, 27]. Socially unacceptable attitudes or actions may also be less likely to be censored on the Internet given perceptions of anonymity, and may in fact serve as an outlet for unpopular beliefs.

An Internet search-based measure of area racism may serve as a more direct indicator of racial attitudes and the extent of discrimination and prejudice towards Blacks in a geographic area, including those experiences of racially motivated bias that are subtle or not observable, and which are not necessarily reported in survey instruments. Accordingly, such measures may improve our ability to investigate racism and help us to examine its consequences for health disparities at the population-level. The purpose of this study was to examine the association between an Internet search-based measure of area racism and Black mortality rates in the US.

## Methods

### Study Design

The unit of analysis in this study is the media market or designated market area (DMA) as defined by Nielsen Media Research. There are a total of 210 DMAs in the US, which are geographic areas receiving common television broadcasts or radio programming; they may also receive other similar media content, such as newspapers or Internet advertising. Hence, although there is heterogeneity within DMAs, they represent a coherent unit of analysis because residents may be exposed to similar social messages influencing racial attitudes.

The main predictor variable of this study is area racism within DMAs [23]. This previously developed variable was calculated as the proportion of total Internet search queries containing the “N-word” (singular or plural) using Google from 2004–2007. More colloquial forms of the “N-word” (i.e., ending in “-a” or “-as”) were not included given prior analysis of top searches revealing that these versions were used in different contexts compared to searches of the term ending in “-er” or “-ers” [23]. Data on area racism were unavailable for Alaska and small DMAs, resulting in a total of 196 DMAs included in analyses. Area racism was standardized so that a one-unit increase in area racism indicates a one standard deviation higher proportion of Google searches containing the “N-word.” Supporting the validity of this measure were statistically significant correlations found with explicit measures of racial attitudes (i.e., a measure of beliefs about interracial marriage included in the General Social Survey) and area-based measures of racial composition (i.e., the percent of Blacks living in an area) [23]. Google query data for this study are available upon request.

Our primary outcome was mortality rates within DMAs for Blacks age 25 and over. Data included information from death certificates and mid-year population counts from 2004–2009 collated by the National Center for Health Statistics (NCHS) [28]. Data are publicly available. Interested researchers can apply to the National Association for Public Health Statistics and Information Systems to access the data at the following website: https://naphsis-web.sharepoint.com/Pages/VitalStatisticsDataResearchRequestProcess.aspx. We examined deaths among those 25 years of age and older, which represent the largest burden of mortality from chronic diseases that are likely to be influenced by social stressors stemming from racism. Across this period, among Blacks there was an average of 23.1 million person-years and approximately 270,000 deaths from all causes each year. For each year, DMA-specific age- and sex-adjusted Black mortality rates weighted using the US 2000 standard population were calculated per 100,000 person years.

We analyzed data on all-cause mortality and the four leading specific causes of death among Blacks identified using International Classification of Disease, Version 10 codes: heart disease (I00-I09, I11, I13, I20-I51); cancer (C00-C97); stroke (I60-I69); and diabetes (E11-E14) [29]. To adjust for relevant area-level covariates, we used data from the American Community Survey (2004–2009), which were merged with and aggregated within DMA-level group identifiers [30]. These data are publicly accessible through the U.S. Census Bureau and can be downloaded at http://www.census.gov/acs/www/data_documentation/data_main/. We examined the following DMA-level covariates: percent of the population that is Black; urbanicity, defined as the percent of people within a DMA living in a city with 50,000 or more people; the percent of Blacks with up to a high school education; and the percent of Black households living in poverty. We also adjusted for all-cause and cause-specific mortality rates among Whites 25 years of age and older, which were calculated using 2004–2009 NCHS data. Using similar methods, age and sex-adjusted mortality rates weighted using the US 2000 standard population were calculated among Whites. White mortality rates per 100,000 person-years were calculated each year at the DMA-level.

### Analysis

Our primary outcomes were derived based on the number of deaths within DMAs and are thus best modeled using analytic methods appropriate for examining count data. Negative binomial regression models were used to examine the association between area racism and Black mortality rates. This was used in lieu of Poisson regression because data are over-dispersed (α>0), which occurs when the variance is larger than the mean [31]. Over-dispersion was evident in all of our models; however, when data are not over-dispersed, results derived from negative binomial and Poisson regression are equivalent. Mid-year population counts were used to model the population at risk of death. Because data include repeated observations within geographic regions, analyses were adjusted at the DMA-level using a Huber-White clustered standard error calculation [32]. For ease of interpretation, mortality rate ratios [MRR], 95% confidence intervals 95 [CI], and p-values are provided. Model fit was assessed using Pseudo-R^2^ and accompanying p-values were calculated using an F-test. To compare models, we further provide the Akaike information criteria (AIC) and calculate the relative likelihood (R_L_), which estimates the probability that a nested model is better than its more parsimonious counterpart [33]. We interpreted an R_L_<0.05 to indicate that the second model is preferable to its comparison. Statistical analyses were done using Stata 13/IC.

We specified four nested models: Model 1 examined the association between area racism and the Black mortality rate; Model 2 further adjusted for overall DMA-level covariates, namely, urbanicity and % Black; Model 3 incorporated race-specific education and poverty among Blacks at the DMA-level; and finally, Model 4 adjusted for the White mortality rate at the DMA-level to further adjust for unmeasured confounders that influence mortality overall in a geographic area, and to examine Black-White disparities in the mortality rate. In models predicting Black mortality rates from heart disease, cancer, stroke, and diabetes, we adjusted for corresponding White cause-specific mortality rates. All models accounted for the decedent’s age (in ten-year groups), sex, year of death, and US Census region of residence (Northeast, Midwest, South, and West), which were recorded from death certificates.

### Ethics Statement

This study involved secondary analysis of existing data and therefore is exempt from human subjects review. Mortality and population data were collated by the National Center for Health Statistics and the US Census Bureau, which are anonymized and publicly available. Google query data were in aggregate at the DMA-level and contain no individual identifiers.

## Results

**Fig 1:**
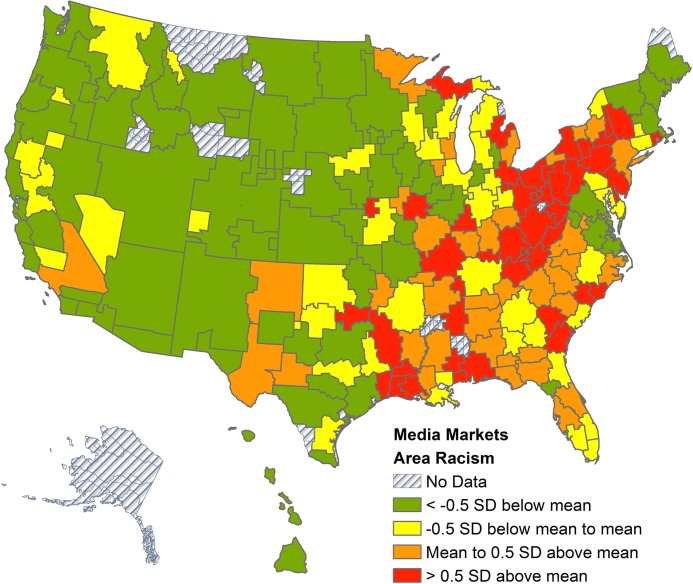
Proportion of Google queries containing the “N-word” by designated market area, 2004–2007.

The distribution of area racism by DMAs is illustrated in Fig 1. Notably, this shows that greater proportions of Google search queries containing the “N-word” were concentrated in the rural Northeast and South of the US.

Descriptive characteristics of variables under investigation are shown in Table 1. On average, these indicate that around 11% of the population aged 25 and over in included DMAs was Black. More than two-thirds of the population lived in or around urban areas. Around 50% were aged 45 and older. Approximately 80% of Blacks had up to a high school degree or equivalent, and almost 30% lived below the poverty line. Across DMAs, Blacks had higher all-cause and cause-specific mortality rates (heart disease, stroke, cancer, and diabetes) compared to Whites. Rates per 100,000 person-years within each of these racial groups are shown in Table 1.

**Table 1:** Descriptive characteristics.

Individual Characteristics		Area Characteristics	
% Aged 45+	49.37%	Urbanicity	66.18%
% Female	52.81%	% Black	11.85%
Year of Death		Education	79.45%
2004	16.55%	Poverty	29.69%
2005	16.87%	Black Mortality rate per 100,000	
2006	16.68%	All-Cause	1765.39
2007	16.66%	Heart Disease	473.30
2008	16.67%	Cancer	262.23
2009	16.57%	Diabetes	19.39
Census Region		White Mortality rate per 100,000	
Northeast	12.93%	All-Cause	1167.29
South	15.54%	Heart Disease	260.16
Midwest	46.87%	Cancer	193.44
West	24.66%	Diabetes	11.11

Note: Among ≥ 25 years of age. Area characteristics at the designated market area level from the American Community Survey, 2004–2009 for urbanicity (% living in a city with ≥ 50,000 people); % Black; education among Blacks (% with up to high school education); and poverty among Blacks (% households in poverty). Race-specific age and sex-adjusted mortality rates weighted using the US 2000 standard population per 100,000 person-years from death certificates and mid-year population counts collated by the National Center for Health Statistics, 2004–2009.

Results from negative binomial regression predicting the all-cause Black mortality rate per 100,000 person years adjusting for age, sex, year of death, and region are shown in Table 2 (Model 1). We found a significant positive relationship between area racism and the Black all-cause mortality rate (MRR = 1.082; 95% CI = 1.056, 1.108; p<0.001). Adding urbanicity and the percent of Blacks significantly improved model fit (Model 2), and attenuated the effect of area racism on the all-cause Black mortality rate (MRR = 1.076; 95% CI = 1.052, 1.10; p<0.001). Adding socioeconomic variables (education and poverty among Blacks) also resulted in a better fitting model (Model 3) and diminished the effect of area racism, but it remained a significant predictor (MRR = 1.057; 95% CI = 1.034, 1.080; p<0.001). Finally, adding the White all-cause mortality rate significantly improved the fit of the model (Model 4). Area racism continued to be significantly associated with the Black all-cause mortality rate (MRR = 1.036; 95% CI = 1.015, 1.057; p = 0.001).

**Table 2:** Nested negative binomial regression models estimating associations with Black all-cause mortality rates.

	Model 1			Model 2			Model 3			Model 4		
	MRR (95% CI)		p	MRR (95% CI)		P	MRR (95% CI)		p	MRR (95% CI)		p
Area racism	1.082 (1.056, 1.108)		<0.001	1.076 (1.052, 1.101)		<0.001	1.057 (1.034, 1.080)		<0.001	1.036 (1.015, 1.057)		0.001
Urbanicity				1.000 (0.999, 1.001)		0.963	1.002 (1.001, 1.004)		0.001	1.004 (1.003, 1.005)		<0.001
% Black				1.006 (1.005, 1.008)		<0.001	1.007 (1.005, 1.009)		<0.001	1.006 (1.004. 1.007)		<0.001
Education							1.003 (0.998, 1.008)		0.199	1.001 (0.996, 1.006)		0.659
Poverty							1.012 (1.008, 1.016)		<0.001	1.010 (1.006, 1.014)		<0.001
White Mortality										1.046 (1.032, 1.059)		<0.001
Psuedo-R^2^		0.309	<0.001		0.314	<0.001		0.317	<0.001		0.321	<0.001
AIC, R_L_		14472	<0.001		14371	<0.001		14294	<0.001		14221	<0.001
Alpha		0.025	<0.001		0.022	<0.001		0.020	<0.001		0.018	<0.001

Note: MRR = Mortality Rate Ratio; CI = confidence interval; AIC = Akaike Information Criteria; R_L_ = Relative Likelihood.

Among ≥ 25 years of age. Race-specific age and sex-adjusted mortality rates weighted using the US 2000 standard population per 100,000 person-years from death certificates and mid-year population counts collated by the National Center for Health Statistics, 2004–2009. Area characteristics at the designated market area level from the American Community Survey, 2004–2009 for urbanicity (% living in a city with ≥ 50,000 people); % Black; education among Blacks (% with up to high school education); and poverty among Blacks (% households in poverty). All models adjusted for individual age, sex, year of death, and Census region.

Also in this final model, increasing age was associated with greater Black mortality rates; for example, with those aged 65–74 (MRR = 17.594, 95% CI = 17.031, 18.176; p<0.001) having greater morality rates compared to those aged 25–34 (the reference). Men also had greater morality rates compared to women (MRR = 1.362; 95% CI = 1.284, 1.444; p<0.001). There was a decrease in the Black mortality rate over time (MRR = 0.980; 95% CI = 0.977, 0.983; p<0.001). Compared to the Northeast, residence in the Midwest (MRR = 1.135; 95% CI = 1.050, 1.227; p = 0.001) and South (MRR = 1.120; 95% CI = 1.040, 1.205; p = 0.003) were associated with a greater Black mortality rate; there was no significant difference in the Black mortality rate associated with living in the West compared to the Northeast (MRR = 1.036; 95% CI = 0.947, 1.149; p = 0.387).

Results from models examining cause-specific mortality rates for leading causes of death among Blacks are summarized in Table 3. These show estimates from models equivalent to final models in Table 2, but adjusting for corresponding White cause-specific mortality rates. Area racism was significantly associated with Black mortality rates for heart disease (MRR = 1.043; 95% CI = 1.015, 1.072; p = .003), cancer (MRR = 1.026; 95% CI = 1.002, 1.050; p = 0037), and stroke (MRR = 1.033; 95% CI = 1.002, 1.065; p = .038). Area racism was not significantly associated with diabetes mortality rates (MRR = 0.946; 95% CI = 0.878, 1.019; p = 0.141).

**Table 3:** Negative binomial regression models estimating associations between area racism and Black cause-specific mortality rates.

	MRR	95% CI	p
Heart Disease	1.043	(1.015, 1.072)	0.003
Cancer	1.026	(1.002, 1.050)	0.037
Stroke	1.033	(1.002, 1.065)	0.038
Diabetes	0.946	(0.878, 1.019)	0.141

Note: MRR = Mortality Rate Ratio; CI = confidence interval.

Among ≥ 25 years of age. Race-specific age and sex-adjusted mortality rates weighted using the US 2000 standard population per 100,000 person-years from death certificates and mid-year population counts collated by the National Center for Health Statistics (NCHS), 2004–2009. All models adjusted for individual age, sex, year of death, and Census region; area characteristics at the designated market area (DMA) level (urbanicity, % Black, % high school education among Blacks, Black poverty rate) from the American Community Survey, 2004–2009; and corresponding DMA-level White cause-specific mortality rates per 100,000 person-years from NCHS.

Sensitivity analyses were conducted examining alternative years of mortality data: 2004–2007, 2005–2008, and 2006–2009. These were conducted in order to examine whether considering shorter four-year consecutive periods of mortality data that were concurrent with the years for Internet query data (2004–2007) or occurred in future years would change our results. The magnitude of associations with area racism for each model when using alternative years of mortality data did not change the MRR by more than 10%. Further, the statistical significance of area racism was the same for Black all-cause mortality in all models, as well as for the same specific causes of death when using the mortality data from 2004–2009.

## Discussion

Results from our study indicate that living in an area characterized by a one standard deviation greater proportion of racist Google searches is associated with an 8.2% increase in the all-cause mortality rate among Blacks. This effect estimate amounts to over 30,000 deaths among Blacks annually nationwide. Although this magnitude was attenuated to 5.7% after adjusting for area-level demographic and socioeconomic covariates, particularly with the inclusion of education and poverty among Blacks, these factors are also influenced by racial prejudice and discrimination and therefore could be on the causal pathway. Area racism was statistically significant even after controlling for White mortality rates (3.6% greater mortality rate). These findings indicate that area racism, as indexed by the proportion of Google searches containing the “N-word”, is significantly associated with not only the all-cause Black mortality rate, but also Black-White disparities in mortality.

As a source of stress experienced across the life-course, racism contributes to “weathering” or accelerated declines in health across biological systems caused by the mobilization of resources to meet environmental demands, thus potentially impacting a range of health outcomes [7, 9]. For example, experimentally manipulated experiences of discrimination have been associated with cardiovascular reactivity, particularly in response to scenarios that were motivationally ambiguous [34]. We found robust associations between area racism and heart disease, cancer, and stroke, leading causes of death among Blacks. Racial disparities in mortality from these diseases may be influenced by racism through biobehavioral channels engaged in the threat response [35]. As a source of chronic psychosocial stress, repeated racism may result in a heighted pro-inflammatory state that can have particularly detrimental consequences for the etiology and progression of cardiovascular and other immune disorders [36]. Studies on discrimination have found evidence for adverse consequences for hypertension, atherosclerosis, and their inflammatory mediators [11–13, 37–40]. A recent study found that racism-related factors may also be associated with accelerated aging at the cellular level [41].

We posit that an Internet search-based measure of racism at the DMA-level reflects the extent of racism in a geographic area, which has been shown to negatively impact health outcomes. Self-reports of discrimination may not capture the major and everyday social insults experienced by Blacks, particularly those without an identifiable perpetrator, or because the perceived reasons for negative treatment may be uncertain [42]. Furthermore, explicit measures of racial attitudes are susceptible to the provision of socially desirable responses or self-censorship in reporting publicly unacceptable beliefs [14]. Area-based measures of segregation and isolation also do not directly assess racial attitudes in a geographic area. Along these lines, Internet-search based proxies of underlying population attitudes may be particularly useful in examining beliefs and actions that are not socially sanctioned, such as those related to racism. Accordingly, such measures may be utilized to examine racism that is systematic, institutionalized, subtle, or otherwise hidden, which may not necessarily be captured in self-report instruments. This measure does not entail that all Internet queries containing the “N-word” are motivated by racist attitudes, or that everyone in a region with a racial bias conducts such searches. Using “big data” and aggregating millions of Internet searches yields a high signal-to-noise ratio despite potential sources of measurement error [23].

There are several caveats to our study to be noted. Given its ecological design, our results are limited in making inferences regarding causality. We cannot conclude that racism at either the individual or area-level necessarily causes deaths among Blacks or contributes to an increased risk of mortality among Blacks in a geographic area. Nevertheless, while these results cannot make such causal conclusions, they do question why the extent of Internet search queries containing the “N-word” is associated with Black mortality rates.

Because of the correlational nature of the data, there are also challenges in interpreting the direction of the associations that we found, i.e., the temporal sequence of our primary exposure and outcomes could not be determined. The mortality data we examined overlaps with the search timeframe of the Internet measure. However, sensitivity analyses revealed minor differences when considering alternative periods of mortality data. Although the effects of racism on health have been shown to accumulate over time, racism can also have more immediate effects on mortality (e.g., discrimination in health care settings) [43]. Racism-related factors impact survival from chronic diseases and increase the risk of acute fatal events such as myocardial infarction [44, 45]. Furthermore, recent analyses suggest that current measures of racial bias are reflections of past negative attitudes and are relatively stable. Data on racial attitudes from 1975–2012 in the General Social Survey indicate that while the absolute level of racial bias changes across states, the relative position of states to each other does not substantially differ (K. Keyes, PhD, analysis of publicly available data, November 2013). Correlations were consistently greater than 0.60 (p<0.01 for all), indicating a high level of stability in the relative rank of a state for racial bias across the last 30+ years. Thus, there is suggestive evidence that the Internet search-based measure of area racism is also likely to be relatively invariant from earlier periods.

There are also limitations inherent in studying cause-specific mortality using death certificates, as was done here [46]. In particular, causes of death are likely to be best identified when the cause is unique and obvious, while the potential for bias increases when comorbidities exist or when the cause of death is occluded by more proximal sources. Specifically, previous research indicates that sensitivity in detecting diabetes-related mortality through death certificates is sub-optimal, which may contribute to the lack of association found between area racism and diabetes mortality rates [47]. Furthermore, post-hoc analyses suggested that this null finding may have been due to competing causes of death among Blacks which may mask an association. However, in order for substantive conclusions on other cause-specific outcomes to be artifacts of measurement error, death certificates would need to be systematically biased towards over-diagnosis of heart disease, cancer, and stroke among Blacks compared to Whites in areas with higher levels of area racism. Although our inability to explore this is a limitation of our study, examining all-cause mortality provides some protection against this potential bias.

Despite these limitations, our results have important implications for methods to measure and monitor racism, and to investigate its impact on mortality. Our findings resonate with those of other research documenting the impact of broader macro-social forces on health outcomes. Previous studies have suggested that political representation, social policies, and high coverage race-related events have an influence on health, mortality, as well as indicators of biological dysregulation among Blacks [48–52]. This emerging body of evidence lends additional credibility to our interpretation of findings. Our study is largely consistent with prior research documenting the detrimental health consequences of discrimination and prejudice, and advances directions for population-level health disparities research through the application of an Internet search-based measure of racism to examine associations with Black mortality.
